# Hoffa’s Fracture with Associated Injuries Around the Knee Joint: An Approach to a Rare Injury

**DOI:** 10.7759/cureus.7865

**Published:** 2020-04-28

**Authors:** Subodh Pathak, Abhijeet Salunke, Shailesh Karn, Harish V K Ratna, Praveen S Thivari, Sarthak Sharma, Sabyasachi Jena

**Affiliations:** 1 Orthopedics, Maharishi Markandeshwar (deemed to be University), Ambala, IND; 2 Orthopedics, Gujarat Cancer Research Institute, Ahmadabad, IND; 3 Orthopedics / Orthopedic Surgery / Orthopedic Oncology, Maharishi Markandeshwar (deemed to be University), Ambala, IND

**Keywords:** hoffa’s fracture, patellar dislocation, intercondylar eminence, supracondylar femur, tibia fracture, patella fracture

## Abstract

Fractures of the distal femur typically occur in the axial and sagittal planes. A Hoffa fracture is a coronal plane fracture of the femoral condyle, which accounts for 8.7% to 13% of distal femoral fractures. It is usually associated with other injuries around the knee joint and hence is often missed. We conducted a comprehensive systematic review of papers published in the English language using PubMed, Web of Science, Scopus, and the Cochrane Database, which reported Hoffa's fracture associated with other injuries around the knee joint. We selected 11 eligible papers for final analysis and review. These papers had 12 patients with Hoffa’s fracture, with associated injuries around the knee joint. The associated injuries with Hoffa’s fracture were in the ipsilateral distal femur, proximal tibia fractures, patellar dislocation, patella fracture, and patellar tendon incarceration. The management principles for Hoffa's fracture with associated injuries around the knee joint are: having a high clinical index of suspicion for these injuries, obtaining all trauma series radiographs and computed tomography of the knee, achieving complete articular incongruity, and restoring the functions of the knee joint.

## Introduction

Hoffa’s fracture is a coronal-oriented fracture of the distal femur with the fracture line extending through the medial condyle, lateral condyle, or bicondylar region [1-3]. This fracture presents commonly as an isolated fracture and, in rare instances, it is associated with other injuries around the knee joint. The presence of Hoffa’s fracture warrants proper clinical evaluation and treatment planning for the patient, as these fractures are associated with high-velocity injuries. These injuries usually affect the younger population after a road traffic accident. The diagnosis of Hoffa’s fracture is challenging and needs strong suspicion. The associated injuries with Hoffa’s fracture are in the ipsilateral distal femur, proximal tibia fractures, patellar dislocation, patella fracture, and patellar tendon incarceration. The trauma series radiographs of the knee joint with anteroposterior, lateral, oblique, and skyline views are important for evaluation. Computed tomography (CT) is an important tool for the assessment of Hoffa’s fracture, as these fractures are often missed on plain radiographic evaluation [3]. Magnetic resonance imaging (MRI) and CT of the knee joint plays a vital role in assessing the articular surface congruity of the distal femur and proximal tibia. Hoffa’s fracture is treated with the principles of treatment of intraarticular fractures, with proper articular surface realignment. Closed reduction of a dislocated patella with Hoffa’s fracture in an emergency room and field triage should be avoided to prevent patellar tendon incarceration, patellar tendon rupture, and osteochondral damage. We have conducted a systematic review of Hoffa’s fracture with associated injures around the joint and have suggested a proper clinical approach for the treatment of these injuries.

## Materials and methods

**Figure 1:**
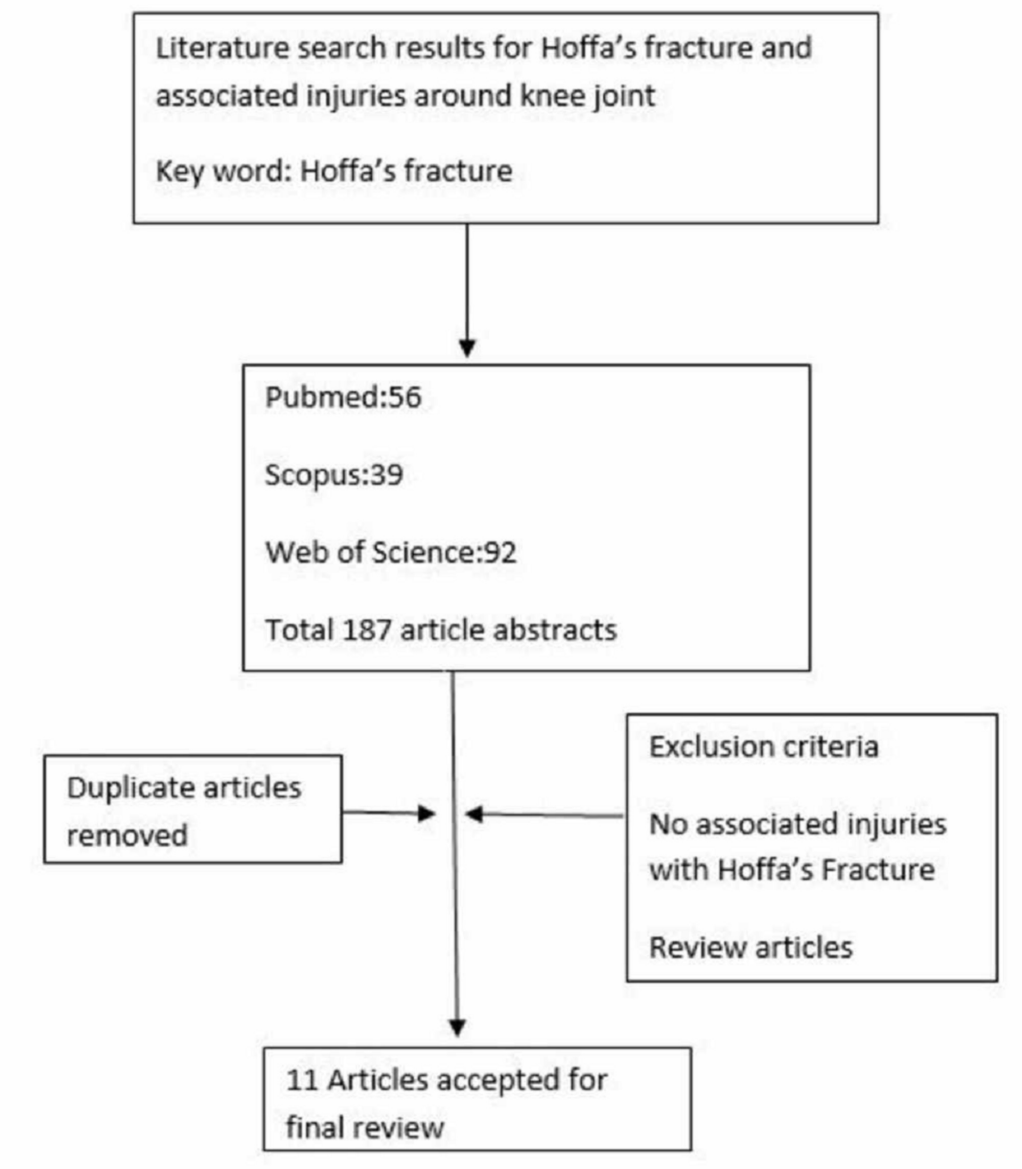
Literature search results for Hoffa’s fracture and associated injuries around the knee joint

We conducted a comprehensive systematic review of papers published in the English language using PubMed, Web of Science, and Scopus, which reported Hoffa's fracture associated with other injuries around the knee joint. Literature search results for Hoffa’s fracture and associated injuries around the knee joint were done with the keyword "Hoffa fracture." Search terms were broad to encompass all possibilities for applicable studies. The search for the studies in the database was performed on February 23, 2017. We did not place any restrictions on the date of publication. Fifty-six articles were identified from PubMed, 92 from Web of Science, and 39 from Scopus after the initial search (Figure 1).

The 187 articles were identified and duplicated articles were removed, and we used the predefined inclusion and exclusion criteria. One-hundred seventy-six articles were excluded on the basis of title and abstract review. We finally selected 11 articles for the systematic review. The supplemental table in the Appendix shows the characteristics of the included studies. The studies that described patients with Hoffa’s fracture and associated injuries around the knee joint were included in this review. The exclusion criteria included animal studies, review articles, pediatric fractures, and Hoffa’s fractures without injuries around the knee joint.

## Results

We selected 11 eligible papers for the final analysis and review. These papers had 12 patients with Hoffa’s fracture, with associated injuries around the knee joint. The associated injuries with Hoffa’s fracture are femoral shaft fractures, supracondylar femur fractures, knee dislocations, patellar tendon incarcerations, patella fractures, and patellar dislocation (as given in the supplemental table in the Appendix). Out of the 12 patients, 50% (n=6) patients had an injury to the patella or the extensor mechanism, making it the most common associated injury. The other common associated injury was a femoral shaft fracture, occurring in 33.34% (n=4).

Baker et al. in 2002 described a case of unicondylar Hoffa’s fracture with a supracondylar fracture of the femur after a road traffic accident [4]. Calment et al. presented a case series of two cases of open bicondylar Hoffa’s fracture with associated injuries around the knee joint following a road traffic accident [5]. The first case was a patellar tendon rupture with Hoffa’s fracture and the patient was treated with fixation of the patellar tendon with non-absorbable suture material. The second case was a distal femur fracture and quadriceps tendon rupture and the patient was treated with cancellous screw fixation and fixation of the quadriceps tendon with non-absorbable suture material. Miyamoto et al. and Gong et al. described a case of a femoral shaft fracture with unicondylar Hoffa’s fracture and the patient was treated with interlocking nailing and internal fixation, respectively [6-7]. Hoffa’s fracture with associated tibia spine avulsion and posterior cruciate ligament rupture was treated with suture anchors for PCL fixation [8]. Bali et al. described a case of tibia spine avulsion and quadriceps tendon rupture and was treated with non-absorbable suture material [9]. A patella fracture with associated Hoffa’s fracture is treated with tension band wiring [10-13]. Shetty et al. described a case of patellar tendon incarceration in Hoffa’s fracture with an associated patellar fracture [10]. Vaishya et al. described a case of patellar dislocation with Hoffa’s fracture following a household traumatic event [14]. In this case report, the diagnosis of Hoffa’s fracture was missed at the initial stage in the primary hospital and a closed reduction of patellar dislocation was performed, resulting in an avulsion patellar fracture. Subsequently, an open reduction of Hoffa’s fracture was performed with lateral patellar retinaculum repair. Hoffa’s fracture with femur shaft and tibia fracture was described by Jain et al. and the patient was treated with internal fixation [15]. Femoral-sided fracture and dislocation of the knee joint were described by Schenck et al. [16]. Nietosvaara et al. described the association of osteochondral fractures with patellar dislocation [17]. Acute dislocations of the patella without associated injuries are treated with a closed reduction method. The technique of closed reduction of the patella involves two steps, i.e., the gradual extension of the knee joint and manual pressure over the lateral aspect of the patella. Closed reduction of a dislocated patella without proper clinical examination and radiological assessment in the emergency room and field triage is not recommended, as it can lead to patellar tendon incarceration, patellar tendon rupture, and osteochondral damage, patella fracture [14,17-19]. Post-traumatic patella dislocation is associated with osteochondral or chondral injuries [17-19].

## Discussion

A coronal-oriented fracture of the distal femur with the fracture line extending through the femoral condyle was described first in 1904 by Hoffa [1]. In 1946, Smillie et al. described these fractures in the review of injuries around the knee joint [2]. The incidence of Hoffa’s fracture is 0.65% of all femoral fractures [3]. The femoral condyles are anatomically in line with the front of the femoral shaft and project backward beyond the shaft, similar to the shape of the alphabet “J.” The medial condyle is larger, more curved, and projects further than the lateral condyle, accounting for the angle between the femur and the tibia [20]. The medial condyle is inferior to the lateral condyle by an average of 7.14 mm on the right side and 7.06 mm on the left side in relation to the femoral shaft axis [21].

Mechanism of Injury and Classification of Hoffa’s Fracture

The normal riding posture of the motorcyclist involves sitting with the knee flexed at or beyond 90 degrees. The lateral femoral condyle receives a direct impact during road traffic accidents because the knee joint is flexed and abducted while driving a two-wheeler vehicle. The mechanism of injury of Hoffa’s fracture is the direct impact of the flexed knee joint to the ground surface or due to simultaneous vertical shear and twisting forces [6-9]. The majority of these injuries are due to motor vehicle accidents and are rarely associated with trivial injuries [11,22-23]. Hoffa’s fractures are classified as AO type 33 B3 fractures based on partially intra-articular localization of the fracture into unicondylar and bicondylar fractures [24]. The anatomical classification is based on the localization of the fracture line into three types, i.e., lateral Hoffa’s fracture and bicondylar Hoffa’s fracture and medial Hoffa’s fracture. The lateral Hoffa’s fracture and bicondylar Hoffa’s fracture are common as compared to the medial Hoffa’s fracture [11-12]. In 1978, Letenneur et al. described the classification of Hoffa’s fracture based on the distance of the fracture line from the posterior femoral cortex into three types [25].

Clinical Examination and Radiological Evaluation

**Figure 2:**
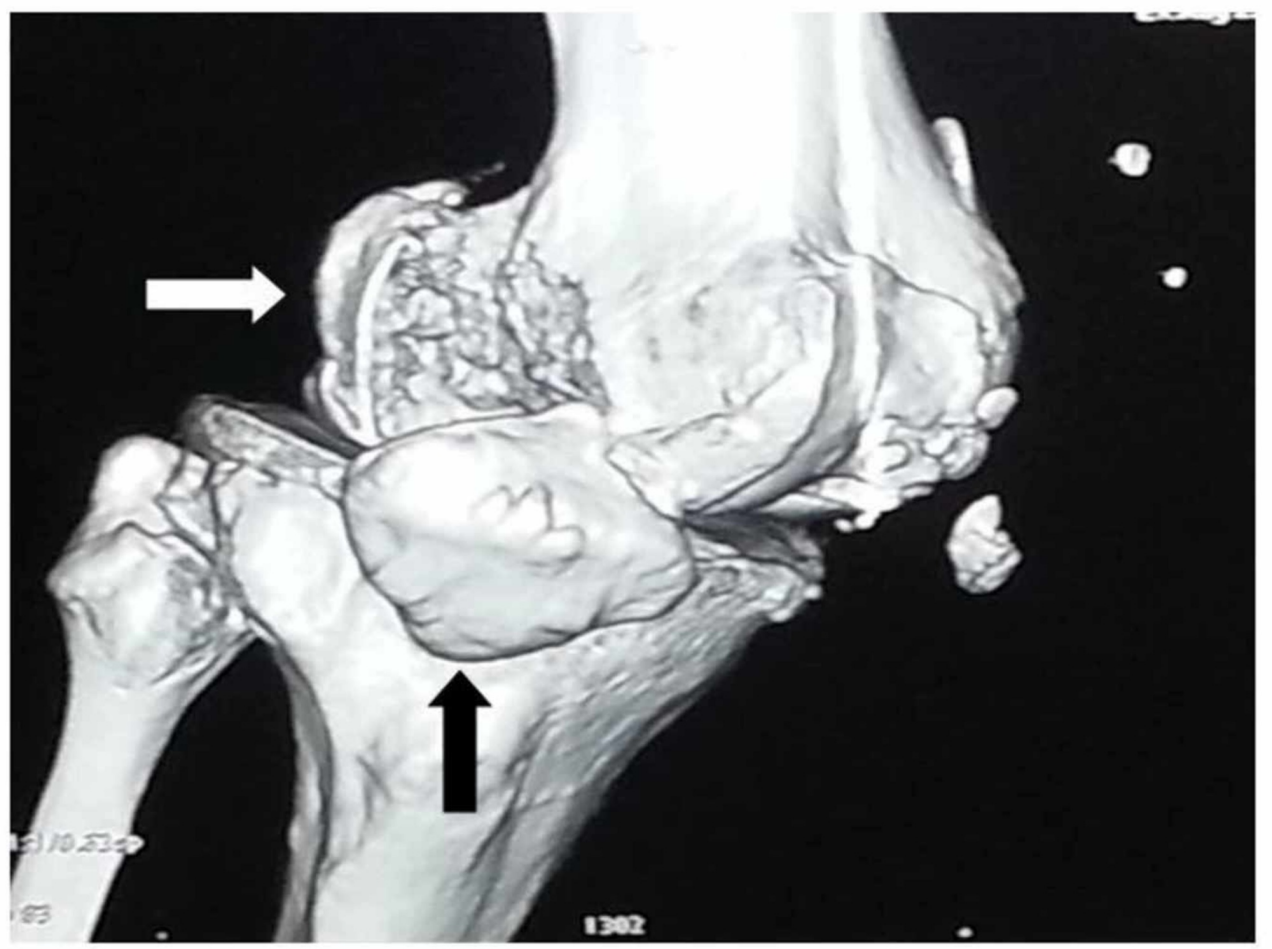
3-D computed tomography showing Hoffa’s fracture (white arrow) with patella dislocation (black arrow)

**Figure 3:**
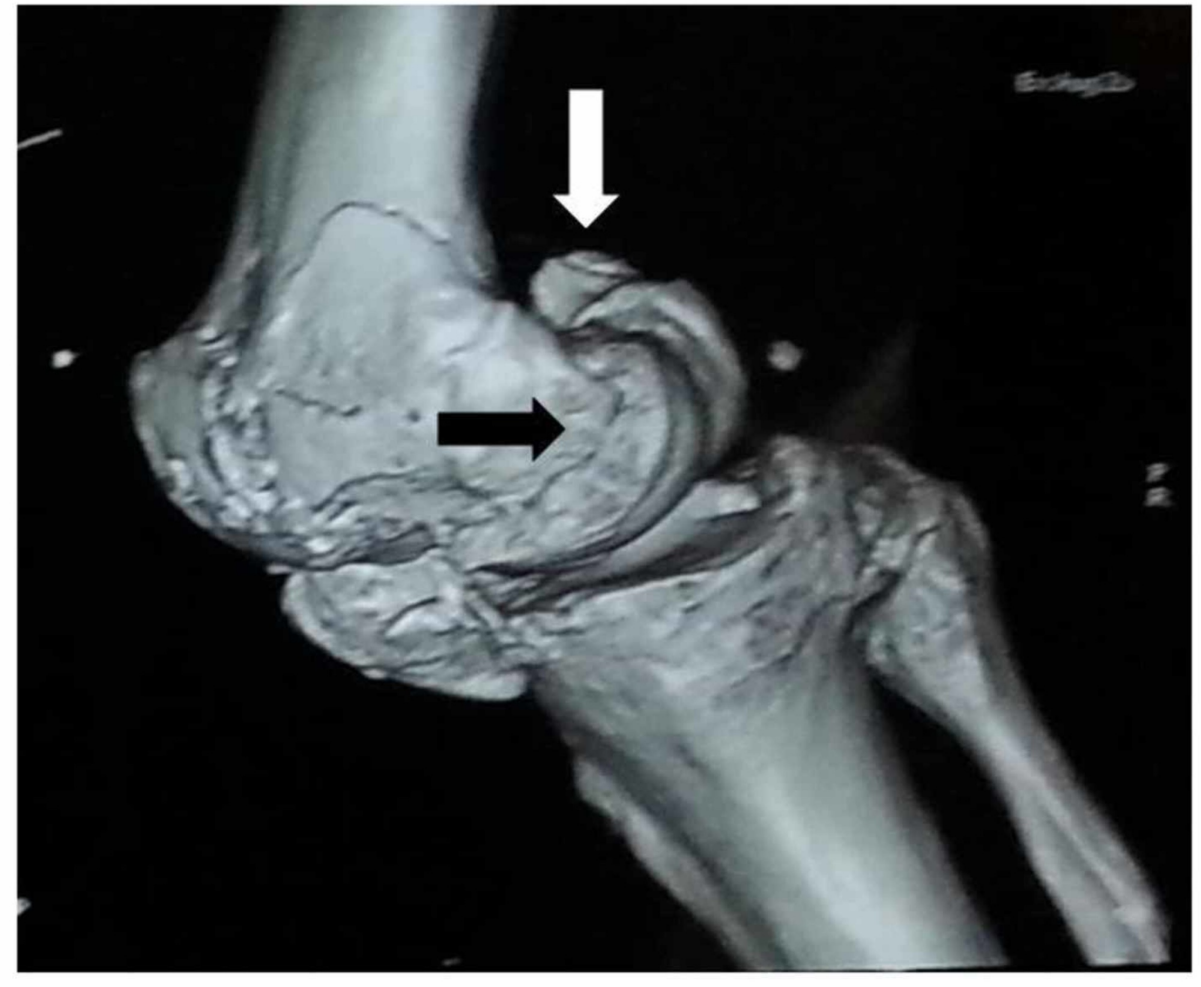
3-D computed tomography showing bicondylar Hoffa’s fracture: medial condyle (black arrow) and lateral condyle fracture (white arrow)

Hoffa's fracture evaluation should be a routine part of the lower-limb and pelvis examination with or without injury [26]. A patient with Hoffa’s fracture presents after sustaining a high-velocity road traffic accident, and rarely due to trivial trauma, with swelling and pain around the knee joint and difficulty in walking. A detailed examination of the affected leg is necessary during the triage and emergency department assessment. The radiographic evaluation includes anteroposterior, lateral, oblique, and tangential patellar and tunnel views for the diagnoses of associated injuries around the knee joint [24-25,27]. The clinical features suggestive of a distal femur fracture are swelling and tenderness around the knee joint. The diagnosis of Hoffa’s fracture is challenging and needs strong suspicion after a clinical examination of the patient. Interpretation of an anteroposterior radiograph of the knee joint is difficult because Hoffa’s fracture is obscured due to the intact anterior part of the femoral condyles [2]. An oblique radiograph is the most important radiograph in the evaluation of Hoffa’s fracture, as it provides proper orientation of the fracture line [24,27]. The radiographs of the femur and tibia must be considered during the evaluation of patients with other suspected injuries. A CT scan of the knee joint provides orientation of the fracture line, with subchondral and intraarticular fracture fragments helping in preoperative planning (Figures 2-3) [24,27].

Magnetic resonance imaging can be used for the assessment of patellar tendon integrity during patellar tendon incarceration into Hoffa’s fracture.

Management

Hoffa’s fractures are difficult to manage because the entire fragment may be covered by articular cartilage and may be devascularized. The surgical approach to these complex fractures and associated injuries and fractures is varied according to the pattern of injuries. Lateral Hoffa’s fractures are approached through a lateral incision; a bicondylar Hoffa’s fracture may require a medial and lateral double incision with tibial tubercle osteotomy [6-9,28-29]. A medial Hoffa’s fracture can be approached through a medial parapatellar arthrotomy or subvastus approach [28,30]. Non-displaced Hoffa’s fractures can be treated non-operatively; displaced fractures require surgical fixation in the form of cancellous lag screws or a buttress plate [27-28]. The screws are inserted down to the subchondral bone well beneath the surface of articular cartilage [27]. Postoperative anteroposterior, lateral, and oblique radiographs of the knee joint are used to assess the screw position and the reduction of the articular congruity of the knee joint. Postoperative management includes proper physiotherapy and knee mobilization exercises to achieve the full range of movement of the knee joint and proper function of the leg. Continuous passive motion exercises could be started on the third postoperative Day 1 to prevent knee stiffness and partial weight-bearing can be started as per the adequacy of surgical fixation and associated injuries. The complications of Hoffa’s fracture are post-traumatic arthritis, axial mal-alignment, instability, and knee stiffness. The complications of this fracture, i.e., malunion and post-traumatic arthritis, can lead to early degenerative changes and loss of function. Osteochondral fragments in the knee joint can cause recurrent patellar dislocation or subluxation [17,19]. A patella fracture is an associated complication due to the closed reduction of the patellar dislocation with Hoffa’s fracture [14].

## Conclusions

Hoffa’s fracture with associated injuries around the knee joint is an uncommon injury that has a high potential for missed diagnosis and improper treatment. An oblique view along with a lateral view is often required to diagnose the injury. The open reduction of patella dislocation, repair of the patellar and quadriceps tendon, fixation of the patella in the anatomical position, with the proper articular reconstruction of Hoffa’s fracture, are the key steps of treatment of this injury.

## Appendices

**Table 1:** Review of literature and study characteristics

Author	Year Publication	Associated injury	Mechanism of Injury	Management	Type of fracture
Baker [4]	2002	Supracondylar femur fracture	Road traffic accident	-	Unicondylar
Calmet [5]	2004	Patella tendon rupture; Distal femur and quadriceps tendon rupture	Road traffic accident; Road traffic accident	Cancellous screw for Hoffa's fracture; Non- absorbable sutures used for fixation of the patellar tendon; Cancellous screw for Hoffa’s fracture; Nonabsorbable sutures used for fixation of the quadriceps tendon	Open fracture Bicondylar; Open fracture Bicondylar
Miyamoto [6]	2006	Ipsilateral femoral shaft fracture	Road traffic accident	Cortical screw for Hoffa’s fracture; Interlocking nailing for femur fracture	Unicondylar
Ocguder [8]	2008	Tibial spine avulsion and PCL rupture	Road traffic accident	Headless cancellous screws for Hoffa’s fracture; Suture anchor for PCL fixation	Unicondylar
Shetty [10]	2008	Patellar tendon incarceration Patella fracture	Road traffic accident	Cancellous screw for Hoffa’s fracture Tension band wiring for patella fracture	Unicondylar
Vaishya [14]	2009	Patella dislocation	Trivial trauma	Emergency patellar reduction Cancellous screw for Hoffa’s fracture Patella fracture treated conservatively	Unicondylar Avulsion patellar fracture
Gong [7]	2011	Femur shaft fracture	Road traffic accident	Cancellous screw for Hoffa’s fracture; Internal fixation of femur fracture	Unicondylar
Jain A [14]	2012	Femur shaft and Tibia fracture	Road traffic accident	Cancellous screw for Hoffa’s fracture Internal fixation of femur and tibia fracture	Unicondylar
Kini [12]	2013	Extensor mechanism	Road traffic accident	Cancellous screw for Hoffa’s fracture; Extensor mechanism repair	Open fracture Bicondylar
Marzouki [13]	2013	Patella fracture	Road traffic accident	Cancellous screw for Hoffa's fracture; Tension band wiring for patella fracture	Unicondylar
Mounasamy [11]	2013	Patella fracture	Road traffic accident	Cannulated, headless screws for Hoffa's fracture; Tension band wiring for patella fracture	Open fracture Bicondylar
